# Identifying an EEG Biomarker for Memory Recall through EEG and EDA in the Presence of Music

**DOI:** 10.1002/alz.085800

**Published:** 2025-01-09

**Authors:** Rupak Kumar Das, Arshia A Khan, Nabiha Zainab Imtiaz

**Affiliations:** ^1^ Pennsylvania State University, State College, PA USA; ^2^ University of Minnesota Duluth, Duluth, MN USA; ^3^ University of Minnesota, Minneapolis, MN USA

## Abstract

**Background:**

There is ample evidence that music can boost brain activity and jog deeply embedded memories. Literature indicates a significant improvement in autobiographical memory (ABM) recall for different individuals during background music sessions. Existing research is based solely on qualitative data, although music has a significant impact on physiological activity. Thus, it's important to explore the connection between memory recall and physiological activities.

**Method:**

To better understand memory recall, the electroencephalogram (EEG) and electrodermal activity (EDA) data were gathered from healthy participants using wearable sensors. Physiological signals such as the electroencephalogram (EEG) and electrodermal activity (EDA) were recorded as quantitative data using various wearable sensors from 40 participants of different age groups while playing different background music sessions. The study involved listening to nine music sessions (three happy, three sad, and three neutral). Immediately after each piece of music, a post‐study survey was conducted to gauge if the participants recalled any autobiographical memories. A machine learning algorithm was developed to train a model using features collected from physiological data to determine if the memory recall was successful. The purpose of the study was to identify an EEG biomarker.

**Result:**

The results of the EEG and EDA data analysis revealed that for all four EEG channels, there was a consistent increase in the alpha power (on average 16.2%) during the memory “recall” scenario (F3: *p* = 0.0066, F7: *p* = 0.0386, F4: *p* = 0.0023, and F8: *p* = 0.0288) compared to the “no‐recall” control. There was also a significant surge in the Beta power for two channels (F3: *p* = 0.0100 and F4: *p* = 0.0210) but not for the control (F7: *p* = 0.6792 and F8: *p* = 0.0814). Additionally, the EDA data analysis revealed significant differences in the phasic standard deviation (*p* = 0.0260), phasic max (*p* = 0.0011), phasic energy (*p* = 0.0478), tonic min (*p* = 0.0092), tonic standard deviation (*p* = 0.0171), and phasic energy (*p* = 0.0478). This implies that the memory recall biomarker is alpha power (8–12 Hz).

**Conclusion:**

The results indicate that the biomarker for memory recall is alpha power (8‐12Hz).

